# Stigmas of holoparasitic *Phelipanche arenaria* (Orobanchaceae) – a suitable ephemeric flower habitat for development unique microbiome

**DOI:** 10.1186/s12870-023-04488-1

**Published:** 2023-10-11

**Authors:** Karolina Ruraż, Sebastian Wojciech Przemieniecki, Magdalena Błaszak, Sylwia Dagmara Czarnomska, Ireneusz Ochmian, Renata Piwowarczyk

**Affiliations:** 1 Center for Research and Conservation of Biodiversity, Department of Environmental Biology, Institute of Biology, Jan Kochanowski University, Uniwersytecka 7, 25-406, Kielce, Poland; 2 Department of Entomology, Phytopathology and Molecular Diagnostics, University of Warmia and Mazury in Olsztyn, Prawocheńskiego 17, 10-720 Olsztyn, Poland; 3 Department of Bioengineering, West Pomeranian University of Technology in Szczecin, Słowackiego 17, 71-434 Szczecin, Poland; 4 Museum and Institute of Zoology, Polish Academy of Sciences, Nadwiślańska 108, 80-680 Gdańsk, Poland; 5 Department of Horticulture, West Pomeranian University of Technology in Szczecin, Słowackiego 17, 71-434 Szczecin, Poland

**Keywords:** Flower microbiome, Parasitic plants, Environment, 16S rRNA gene, Internal transcribed spacer (ITS), Next-generation sequencing

## Abstract

**Background:**

Microbial communities have occasionally been observed in part of the ephemeric reproductive structure of floral stigmas, but their prevalence, phylogenetic diversity and ecological roles are understudied. This report describes the first study of bacterial and fungal communities in immature and mature stigma tissue of the endangered holoparasitic plant *Phelipanche arenaria*. Culture-dependent methods coupled with next-generation sequencing indicated that a small surface of the flower stigma was an unexpectedly rich and diverse microhabitat for colonization of microbial. We also compared the enzymatic activity of the bacterial communities between immature and mature stigmas samples.

**Results:**

Using high-throughput sequencing methods, we identified and classified 39 to over 51 OTUs per sample for bacterial OTUs represented by *Pantoea agglomerans* and *P. ananatis*, comprising 50.6%, followed by *Pseudomonas*, *Luteibacter* spp., *Sphingomonas* spp. with 17% of total frequency. The bacterial profile of immature stigmas of *P. arenaria* contained unique microorganisms (21 of the most numerous OTUs) that were not confirmed in mature stigmas. However, the enzymatic activity of bacteria in mature stigmas of *P. arenaria* showed more activity than observed in immature stigmas. In the fungal profile, we recorded even 80 OTUs in mature stigmas, consisting of Capnodiales 45.03% of the total abundance with 28.27% of frequency was created by *Alternaria eichhorniae* (10.55%), *Mycosphaerella tassiana* (9.69%), and *Aureobasidium pullulans* (8.03%). Additionally, numerous putative plant growth-promoting bacteria, fungal pathogens and pathogen-antagonistic yeasts were also detected.

**Conclusions:**

Our study uncovered that *P. arenaria* stigmas host diverse bacterial and fungal communities. These microorganisms are well known and have been described as beneficial for biotechnological and environmental applications (e.g., production of different enzymes and antimicrobial compounds). This research provided valuable insight into the parasitic plant–microbe interactions.

**Supplementary Information:**

The online version contains supplementary material available at 10.1186/s12870-023-04488-1.

## Background

The stigma is a specialised tissue with a key structure in plant reproduction and an ephemeral nature. Stigmas, along with other structures of flowers, are transient organs and normally are classified into two groups: wet and dry stigmas. This division depends on whether they possess a surface secretion [1, 2]. These are female tissues with receptive portions that intercept pollen and initiate pollen tube growth towards the ovary. Of many different flower organs, stigmas with colour and structure modifications are attractive to pollinators and contribute to pollination success [3–5]. Therefore, the stigma as a part of the gynoecium plays a key role in pollination, fertilization and seed set. These reproductive organs of plants are nutrient-rich and contain proteins, lipids, polysaccharides and other ingredients; thus, they are an important nutrient source for microorganisms [2, 6]. Stigmatic exudates show significant activity in terms of the number of processes and functions in which they participate [7, 8].

Investigations and interest in flower-associated microbiota are related to whole flower analysis and individual parts or structures (nectar, petal, pistil, stigma, pollen and seeds) [9–12]. Many studies have been published in the last years mainly focused on nectar microbial communities and comparison of microbial communities in different floral organs and floral rewards (pollen, nectar) [13–17]. The plant species and pollination type significantly influenced the structure and diversity of the pollen microbiota. The insect-pollinated species possessed more similar microorganisms in comparison to the wind-pollinated ones [16]. Yeasts occurred regularly in the floral nectar (between 32 and 44% of all nectar samples contained yeasts) and their abundance in samples were directly correlated with the proportion of pollinators [18]. However, compared to other flower structures, the analysis of the microbial communities in the stigmas is very poorly understood, especially when it comes to rare plant species. It is possible that microbiota also support plant growth and/or plant development, and reducing disease, and in conferring increased survival, as well as contribute to the adaptation of rare and endangered plants to the environment through nitrogen fixation, phosphate solubilization, or phytohormone production [19–21]. Without this research it is difficult to validate what role these microorganisms can play in the biology of these plant species. In addition, few studies indicate that it is possible to use microbes to protect endangered plant populations in the field, which in the future may help protect them from the extinction [19].

One of the most important stigma-infecting microorganisms in apple and pear diseases is the bacterium *Erwinia amylovora*, which causes fire blight [22–24]. The occurrence and development of pathogens lead to impaired morphology of female reproductive organs and, as a consequence, to failure of sexual reproduction (even shifts in sex ratios to nearly 100% phenotypically hermaphroditic) [25–27]. Some of them colonize stigmas as a pathway to infect seeds (e.g., *Acidovorax citrulli* in cucurbits, *Monilinia vaccinii-corymbosi* on blueberry) [28, 29]. However, little is known about how stigma microbial communities can influence disease and reproductive success of plants. It may also depend on the analyzed plant species and climatic conditions (mainly temperature and humidity). Research has demonstrated that microorganisms are transferred from blossoms to fruits, and this transfer is most pronounced during the growth and maturation of the fruit [30]. The microbiome present in flowers has an impact on floral rewards, which are significant for pollination and, consequently, for the reproductive process [31]. The results of these studies may provide a promising prospect for the biological control of diseases, because microorganisms associated with flowers are known to be related to plant health and fitness [32, 33].

For our research, we chose *Phelipanche arenaria* (Borkh.) Pomel (syn. *Orobanche arenaria* Borkh.), sand broomrape, an obligate parasite belonging to the family Orobanchaceae. Orobanchaceae is the largest parasitic flowering plant family, with 102 genera and over 2100 species [34]. The main lineages of Orobancheae are widely distributed, with the main centres of their biodiversity and origin being the Mediterranean Basin and Western Asia [35]. Holoparasitic *Orobanche* s.l. species (broomrapes) are chlorophyll-lacking root parasites of cultivated crops and wild plants. However, the majority of species are rare and threatened with extinction. *Orobanche* L. and *Phelipanche* Pomel are the largest root-holoparasitic genera and together comprise ca. 200 species. Orobanchaceae is one of the most critical families of flora worldwide and is notoriously difficult to determine. This is caused by greatly reduced vegetative organs and high morphological variability, which is associated with their heterotrophic lifestyle [36, 37].

Previous studies have documented the microbial diversity of parasitic plants in relation to their negative effects on host crop plants [38–40]. However, most of them comprise species that are rare and endangered, as well as keystone components of natural ecosystems [41]. The occurrence of endophytic microbiota in seeds has a functional effect on the specialized seed germination mechanisms of parasitic species with their hosts (*P*. *ramosa* (L.) Pomel vs. tomato, rape, tobacco, and hemp) [39]. These microorganisms were recorded as plant growth-promoting species and resistant to environmental conditions [39, 40, 42]. Moreover, an exchange of endophytes between the parasite and the host showed that the parasitic plant microbiome is obtained but distinct from the host plant microbiota (*P*. *aegyptiaca* (Pers.) Pomel vs. tomato and *O*. *hederae* Vaucher ex Duby vs. ivy) [38, 43]. Higher humidity and a rich source of nutrients are important habitats for microorganisms (bacteria and yeasts) in the floral nectar of *P. ramosa* and *O. rapum-genistae* Thuill [15]. The investigation of microbial communities in parasitic plants with their hosts is an alternative and environmentally friendly method for parasitic weed control. Parasites can be considered keystone species because they can influence not only individual species’ growth, reproduction or allometry but also ecosystems or communities, such as ecosystem engineers, through their effects on abiotic environments and trophic interactions with other organisms [44] (e.g., pollinators, herbivores, microorganisms, nonhost plants).

The aim of this study was to describe the bacterial and fungal communities of *P. arenaria* stigmas using molecular and culture methods. Moreover, these microbial communities were sampled from immature stigmas from closed flower buds and mature stigmas from opened flowers to study how they change during flower development and exposure to the environment. Additionally, these data shed light on how plant-associated microbiota are formed through the unique ecology and heterotrophic life strategy of parasitic plants. We also discussed about the potential roles of the recorded microorganisms. To the best of our knowledge, this is the first attempt to characterize microbial communities of stigmas of parasitic plant species using both molecular and culture methods.

## Results

### The characteristic of bacteria and fungi isolated from plant tissue

**Fig. 1:**
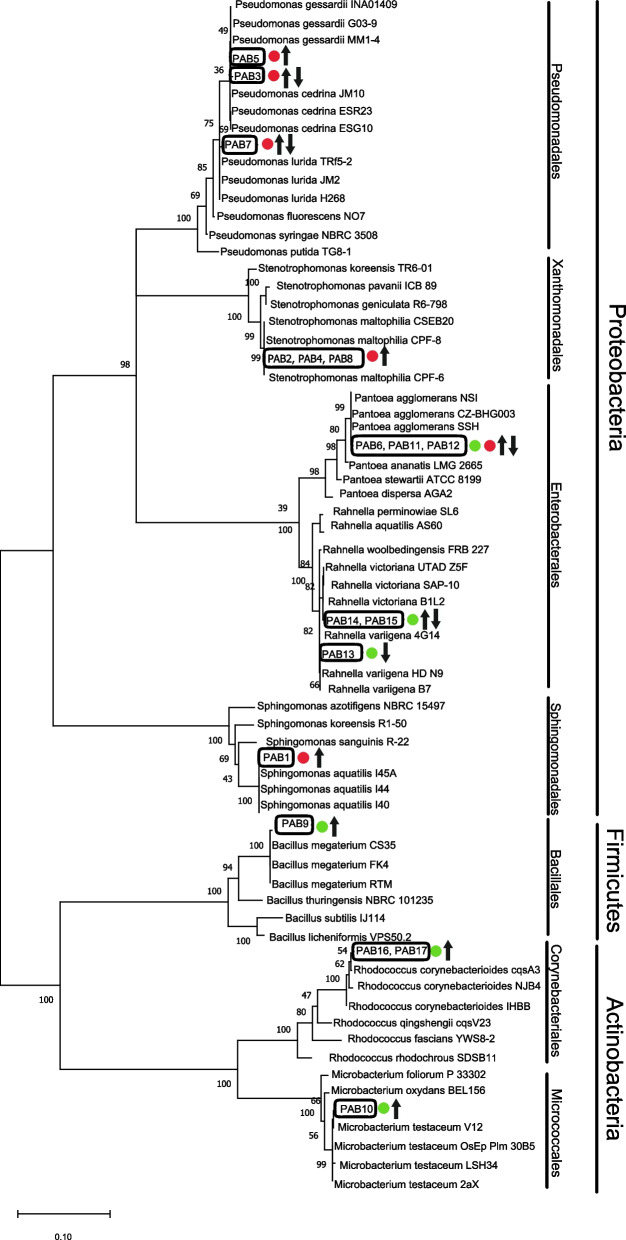
A phylogenetic tree based on 16S rRNA sequences demonstrated for the identified strains of bacteria compared to other strains. The bar indicates sequence divergence. The black rectangle marks bacteria that colonized immature stigmas from closed flowers (green dots) and mature stigmas from opened flowers (red dots). The potentially pathogenic (down arrow) and beneficial effect (up arrow) of microorganisms on plants were presented. PAB – identified strains of bacteria

**Fig. 2:**
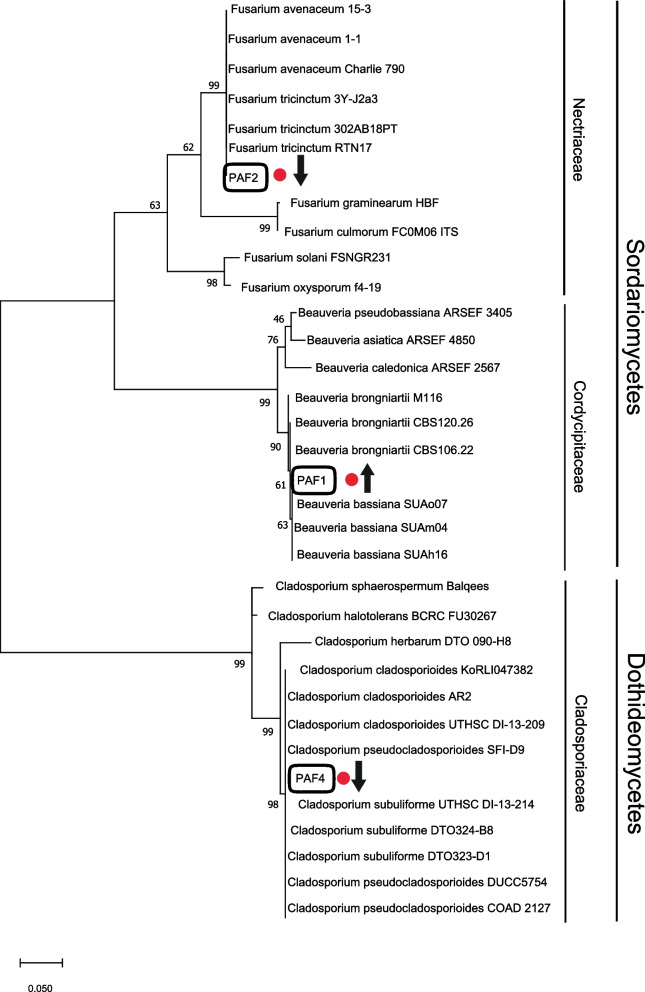
A phylogenetic tree based on ITS sequences demonstrated for the identified strains of fungi compared to other strains. The bar indicates sequence divergence. The black rectangle marks fungi that colonized mature stigmas from opened flowers (red dots). The potentially pathogenic (down arrow) and beneficial effect (up arrow) of microorganisms on plants were presented

**Fig. 3:**
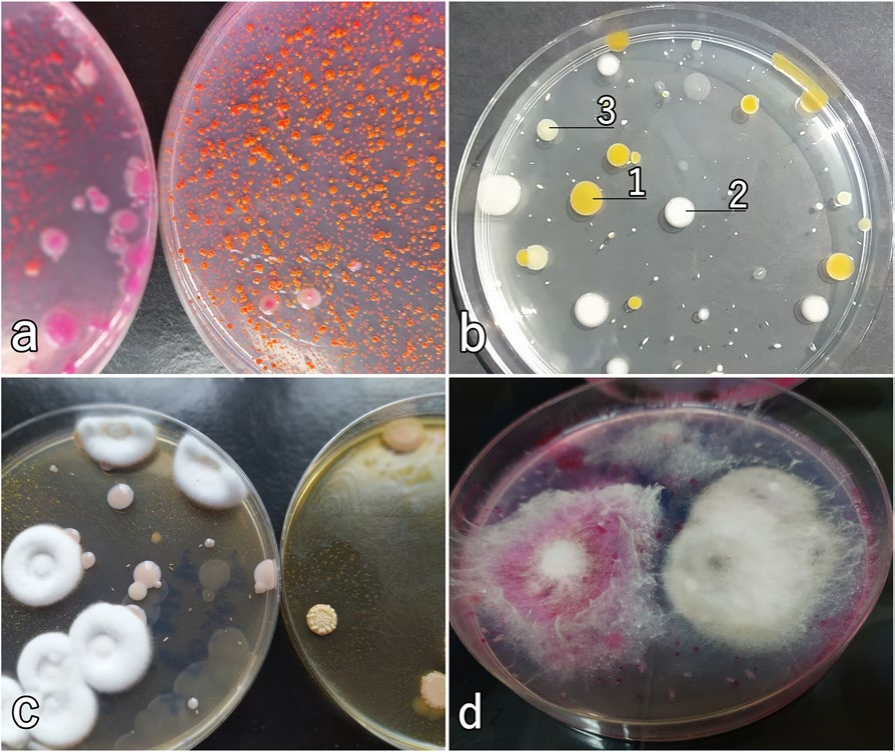
Selected colonies of culturable bacteria and fungi. **a** orange colonies of *Sphingomonas aquatilis* (Rose Bengal Agar), **b** bacterial isolates grown on the Trypticase Soy Agar, dominant: 1. *Pantoea agglomerans*. 2*. Pseudomonas lurida.* 3*. Stenotrophomonas maltophilia*, **c** *Beauveria bassiana* grown on Yeast Extract–Peptone–Glycerol Agar, with *Stenotrophomonas maltophilia* bacteria nearby, **d** *Fusarium avenaceum* (on the left) and *Cladosporium* sp. (on the right) on the Rose Bengal Agar

As a result of inoculation dilutions of the stigmas' suspension on microbiological media, mixed cultures of microorganisms were obtained. To eliminate the repetition of the same strains in the molecular identification kit, the isolates were subjected to the microscopic observation of cells/spores and the observation of colony morphology. Based on morphology and microscopic features, nine bacterial isolates of immature stigmas were selected and identified with Sanger sequencing: *Bacillus megaterium*, *Microbacterium testaceum*, *Pantoea agglomerans*, *Rahnella victoriana*,* R. variigena*, *Rhodococcus corynebacterioides*. Moreover, we recorded the eight bacterial and three fungal isolates of mature stigmas, which were the most numerous in mixed culture and were morphologically different from each other. These strains were isolated and identified: *P. agglomerans*, *Pseudomonas cedrina*, *P. gessardii*, *P. lurida*, *Sphingomonas aquatilis*, *Stenotrophomonas maltophilia*, *Beauveria bassiana*, *Fusarium avenaceum*, *Cladosporium subuliforme* (Figs. 1 and 2). Bacteria belonging to the genera *Pantoea* (yellow colonies), *Pseudomonas* and *Stenotrophomonas* grew on TSA and YPG culture medium (Fig. 3b). Orange colonies of *Sphingomonas aquatilis* dominated on Rose Bengal Agar (Fig. 3a). The *Beauveria bassiana* fungus (Fig. 3c), which did not grow on other media, grew profusely on YPG. Specimens representing the species *Fusarium avenaceum*, and *C. subuliforme* grew on Rose Bengal Agar (Fig. 3d). The total number of bacteria inhabiting the mature stigmas was 5.84 ± 0.02 log CFU·g^−1^ and fungi 3.94 ± 0.03 log CFU·g^−1^ (mean ± SD) while on the immature stigmas was 5.14 ± 0.03 log CFU·g^−1^ bacteria, fungi were absent.

### The characteristic of stigmas microbial communities

Quality filtering recovered a total of 61 578 16S rRNA reads of bacteria and 13 863 ITS reads of fungi from stigma samples. The microbiota analysis of the examined stigma allowed us to identify 39 to over 51 OTUs per sample for bacteria and similar value was recorded between immature and mature stigmas (41 vs. 39). In contrast to the fungal community colonizing the stigmas, where only two OTUs were recorded in immature stigmas, however in mature stigmas observed even 80 OTUs (Table 1).

**Table 1:** OTUs and diversity indices of microbiota identified in *Phelipanche arenaria* (PA) stigmas

Measure	**PA1**^a^	**PA2**^b^	**PA3**^b^	**PA4**^b^
Bacteria				
OTUs	39	51	28	43
Simpson’s dominance (λ)	0.39	0.16	0.44	0.19
S-W diversity (H′)	1.57	2.67	1.48	2.56
Pielou’s evenness (J′)	0.43	0.68	0.45	0.68
Fungi				
OTUs	80	2	-^c^	-
Simpson’s dominance (λ)	0.23	0.78	-	-
S-W diversity (H′)	2.23	0.38	-	-
Pielou’s evenness (J′)	0.51	0.54	-	-

^a^mature stigmas collected from opened flowers

^b^immature stigmas collected from closed flowers

^c^in PA3 and PA4 samples fungi was not identified

Analysis of the composition of bacteria of stigmas allowed us to identify bacteria representing 34 most numerous OTUs (Table 2), such as eudominant *Pantoea agglomerans* and *P. ananatis*, comprising 50.6% of the total amount. The next most abundant OTUs ranked were *Pseudomonas*, *Luteibacter* spp., and *Sphingomonas* spp. with 17% of total frequency. The bacterial communities that colonized the stigmas at different stages of development show differences within the eudominats. *P. agglomerans* predominates in immature stigmas, while *P. ananatis* in mature stigmas. In addition, the bacterial profile of immature stigmas of *P. arenaria* contained unique microorganisms (21 of the most numerous OTUs) that were not identified in mature stigmas sample, e.g.: *Luteibacter* spp., *Rhodococcus corynebacterioides*, *Serratia* spp., and *Pseudomonas rhizosphaerae*. However, in mature stigmas were recorded only *P. ananatis*, *P. fragi*, *Xanthomonadaceae*, *Rhizobiaceae* and *Paenibacillus* spp.. Additionally, we observed that during the development of stigmas, some frequency of bacteria decreased in mature stigmas e.g.: in *P. agglomerans* (from 47.43 to 0.06%), *Sphingomonas* spp. (5.36 vs. 0.27%), *Chryseobacterium* spp. (2.01 vs. 0.04%), as well as higher amounts reported in *Pseudomonas* spp. (from 5.09 to 12.70%), Enterobacteriaceae (0.68 vs. 5.67%), *Stenotrophomonas* spp. (0.07 vs. 5.74%), and *Pedobacter* spp. (0.06 vs. 2.55%).

**Table 2:** Frequency (%) of bacteria identified in *Phelipanche arenaria* (PA) stigmas

ID	**PA1**^a^	**PA2**^b^	**PA3**^b^	**PA4**^b^	**Mean**
s__Pantoea agglomerans	0.06*	36.73*	65.44*	40.12*	35.6
s__Pantoea ananatis	60.15*	0.00*	0.00*	0.00*	15.0
g__Pseudomonas	12.70*	7.18	3.69*	4.39*	7.0
g__Luteibacter	0.00*	9.62*	2.93	11.12*	5.9
g__Sphingomonas	0.27*	7.83*	1.57	6.68*	4.1
s__Rhodococcus corynebacterioides	0.00*	1.55	7.42*	2.20	2.8
g__Serratia	0.00*	0.00	7.95*	0.00	2.0
f__Enterobacteriaceae	5.67*	0.00*	2.04	0.00*	1.9
s__Pseudomonas rhizosphaerae	0.00	2.12	1.09	2.97	1.5
g__Chryseobacterium	0.04	3.51	0.00	2.52	1.5
g__Stenotrophomonas	5.74*	0.00*	0.20*	0.00*	1.5
g__Rhodococcus	0.00	1.29	2.22	2.06	1.4
g__Pseudarthrobacter	0.00	2.57	0.00	2.47	1.3
g__Methylobacterium-Methylorubrum	0.00	2.39	0.22	2.42	1.3
g__Duganella	0.00	3.24	0.00	1.10	1.1
g__Microbacterium	0.00	0.88	1.24	1.56	0.9
g__Massilia	0.00	1.64	0.02	1.56	0.8
s__Pseudomonas fragi	2.98	0.00	0.00	0.00	0.7
g__Variovorax	0.00	1.18	0.41	1.24	0.7
g__Pedobacter	2.55	0.19	0.00	0.00	0.7
f__Xanthomonadaceae	2.73	0.00	0.00	0.00	0.7
g__Aeromicrobium	0.00	1.15	0.41	0.96	0.6
s__Pseudomonas tolaasii	0.00	0.75	0.00	1.65	0.6
g__Aureimonas	0.00	0.97	0.35	1.05	0.6
f__Rhizobiaceae	1.94	0.00	0.00	0.00	0.5
g__Xylophilus	0.00	0.88	0.00	1.01	0.5
f__Microbacteriaceae	0.09	1.10	0.00	0.64	0.5
g__Blastococcus	0.00	1.66	0.00	0.00	0.4
g__Nocardioides	0.00	1.05	0.00	0.37	0.4
g__Enhydrobacter	0.00	0.19	0.00	1.14	0.3
s__Lactobacillus curvatus	0.00	0.21	0.00	1.10	0.3
g__Skermanella	0.00	1.18	0.00	0.09	0.3
g__Corynebacterium	0.00	0.00	0.00	1.24	0.3
g__Paenibacillus	1.09	0.00	0.00	0.00	0.3
Eudominant	Dominant	Subdominant	Rare	Occasional	Casual
> 35.01	10.01–35	5.01–10	2.01–5	1.01–2	< 1%

^a^mature stigmas collected from opened flowers

^b^immature stigmas collected from closed flowers

^*^Fisher's exact test (*p*-values), only OTUs with significant biological differences (*P* < 0.05) were shown

In this study, the fungal composition of associated with stigmas of *P. arenaria* is more diverse that the bacterial, although in immature stigmas of *P. arenaria* contained only two OTUs – *Blumeria* spp. (87.5% of the total) and *Alternaria* spp. (12.5%). Thus, the fungal profile of mature stigmas includes a series of microorganisms – 16 most numerous OTUs (Table 3), where the eudominant is Capnodiales (45.03% of the total abundance). The other 28.27% of frequency was created by *Alternaria eichhorniae* (10.55%), *Mycosphaerella tassiana* (9.69%), and *Aureobasidium pullulans* (8.03%).

**Table 3:** Frequency (%) of fungi identified in *Phelipanche arenaria* (PA) stigmas

ID		**PA1**^a^	**PA2**^b^	**PA3**^b^	**PA4**^b^
o__Capnodiales		45.03*	0.00*	0.00	0.00
g__Blumeria		0.00*	87.50*	0.00	0.00
s__Alternaria eichhorniae		10.55*	0.00*	0.00	0.00
s__Mycosphaerella tassiana		9.69*	0.00*	0.00	0.00
s__Aureobasidium pullulans		8.03*	0.00*	0.00	0.00
p__Ascomycota		4.62*	0.00*	0.00	0.00
s__Sarocladium strictum		3.06	0.00	0.00	0.00
c__Dothideomycetes		2.22	0.00	0.00	0.00
g__Alternaria		2.09*	12.50*	0.00	0.00
s__Filobasidium wieringae		1.57	0.00	0.00	0.00
s__Gibberella tricincta		1.43	0.00	0.00	0.00
o__Pleosporales		1.16	0.00	0.00	0.00
f__Mycosphaerellaceae		0.94	0.00	0.00	0.00
g__Phoma		0.89	0.00	0.00	0.00
g__Chaetosphaeronema		0.84	0.00	0.00	0.00
s__Hannaella coprosmae		0.77	0.00	0.00	0.00
k__Fungi		0.65	0.00	0.00	0.00
Eudominant	Dominant	Subdominant	Rare	Occasional	Casual
> 35.01	10.01–35	5.01–10	2.01–5	1.01–2	< 1%

^a^mature stigmas collected from opened flowers

^b^immature stigmas collected from closed flowers

^*^Fisher's exact test (*p*-values), only OTUs with significant biological differences (*P* < 0.05) were shown

Furthermore, the frequency of bacterial microbiota was significantly different between the immature and mature stigmas in *P. agglomerans* and *P. ananatis* (Fisher's exact test *p* < 0.0001), as well as *Stenotrophomonas* spp. (*p* < 0.014 and *p* < 0.029) of all examined samples. For *Pseudomonas* spp., *Luteibacter* spp., *Sphingomonas* spp., *Serratia* spp., and Enterobacteriaceae statistically significant results were also obtained in most of the analyzed samples (Table 2). Due to the presence of only two fungal taxa (only in immature stigmas PA2 sample) compared to the 17 identified OTUs in the PA1 sample, a large part of the results were statistically significant. Based on Fisher's exact test, it was observed that OTUs below 4.62% were not significantly different from each other. Nevertheless, a very strong *Blumeria* spp. eudominance was noted in the PA2 sample, which was not present in any of the other samples. On the other hand, in immature and mature stigmas there was OTU *Alternaria* sp., other than *A. eichhorniae*, characteristic only for mature stigmas (Table 3).

**Fig. 4:**
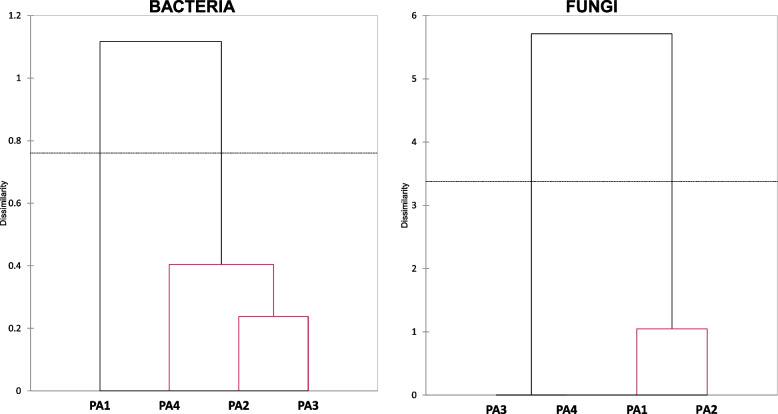
Agglomerative Hierarchy Clustering analysis (Bray‒Curtis method) of distance between phyla and order representing microbial communities inhabiting *Phelipanche arenaria* (PA) stigmas. Immature stigmas from closed flowers (2–4) and mature stigmas from opened flowers (1) (fungi on the right, bacteria on the left)

Agglomerative hierarchical clustering analysis of microbial communities divided the samples of the stigma into two main clades for bacteria and fungi (Fig. 4). The mature stigmas were separated from immature stigmas, suggesting clear distinctions of bacterial community structure among PA1 and PA2-PA4 samples. Contrary to fungi where a sample of mature stigmas (PA1) forms a common group with one sample of immature stigmas (PA2). It should be noted that this connection is due to the presence of only two fungal OTUs in one of the three samples of mature stigmas.

**Fig. 5:**
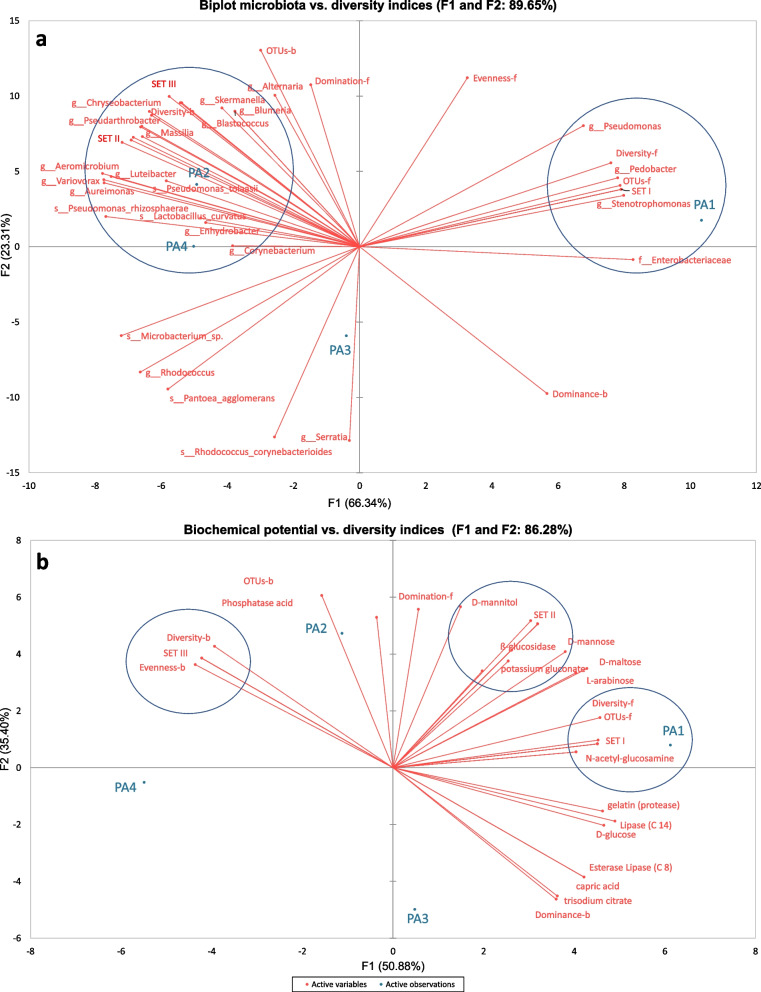
Principal Component Analysis (PCA) of the data on microbial communities and diversity indices (**a**) and the data of biochemical potential of identified microorganisms vs. diversity indices (**b**) of the immature (PA2-PA4) and mature stigma (PA1) *Phelipanche arenaria* samples. The lines represent the correlation coefficient between the principal component scores and each of the factors. **a** Set I included: *Paenibacillus* spp., Rhizobiaceae, Xanthomonadaceae, *Pseudomonas fragi*, *Pantoea ananatis*, *Hannaella coprosmae*, *Chaetosphaeronem*a sp., *Alternaria eichhorniae*, Mycosphaerellaceae, *Gibberella tricincta*, *Filobasidium wieringae*, *Sarocladium strictum*, *Aureobasidium pullulans*, *Mycosphaerella tassiana*, Pleosporales, Dothideomycetes, Ascomycota, Capnodiales, Fungi, *Phoma* sp.. Set II included: *Sphingomonas* spp., *Methylobacterium-Methylorubrum* sp., Evenness-b, *Xylophilus* sp., *Chryseobacterium* spp, *Massilia* sp., *Pseudarthrobacter* sp., Diversity-b, *Duganella* sp., *Nocardioides* sp., Microbacteriaceae. Set III included: *Pseudomonas tolaasii*, *Aeromicrobium* spp., *Luteibacter* spp., *Variovorax* sp., *Aureimonas* spp. **b** Set I included: N-acetyl-ß-glucosaminidase, α-galactosidase, valine arylamidase, leucine arylamidase, malic acid. Set II included: potassium nitrate (nitrate reduction), Naphtol-AS-BI-phosphohydrolase, D-glucose (fermentation), potassium gluconate, ß-galactosidase and evenness indices of fungi. Set III included: cystine arylamidase, phosphatase alkaline and α-glucosidase

By using the principal component analysis (PCA), we decomposed the data on microbial communities and diversity indices into two factors that explained 89.65% of the variance (Fig. 5a). Sample of mature stigmas of *P. arenaria* was correlated with *Stenotrophomonas* spp., *Pseudomonas* spp., *P. fragi*, *Pantoea ananatis*, *Xanthomonadaceae*, *Rhizobiaceae*, *Pedobacter* spp. and *Paenibacillus* spp. with diversity and OTUs indices of fungi. These stigmas were correlated negatively with Enterobacteriaceae. Unlike samples of immature stigmas were influenced with several bacterial genera: *Luteibacter*, *Sphingomonas*, *Chryseobacterium*, *Pseudarthrobacter*, *Methylobacterium-Methylorubrum*, *Duganella*, *Massilia*, *Variovorax*, *Aeromicrobium*, *Aureimonas*, *Xylophilus*, *Microbacteriaceae*, *Blastococcus*, *Nocardioides*, *Enhydrobacter*, *Skermanella*, and *Corynebacterium*, as well as *Pseudomonas tolaasii*, *P. rhizosphaerae*, and *Lactobacillus curvatus* with fungal genus—*Blumeria* and diversity and evenness indices of bacteria. Furthermore, due to the presence of only two OTUs of fungal microorganisms observed in immature stigmas, the PCA showed correlation with all numerous OTUs recorded in mature stigmas (Fig. 5a). Moreover, we decomposed the data of biochemical potential of identified microorganisms vs. diversity indices into two factors that explained 86.28% of the variance (Fig. 5b). The mature stigmas were strongly correlated with N-acetyl-glucosamine, N-acetyl-ß-glucosaminidase, α-galactosidase, valine arylamidase, leucine arylamidase and malic acid with diversity and OTUs indices of fungi. Additionally, the biochemical factors like, D-mannitol, potassium nitrate (nitrate reduction), Naphtol-AS-BI-phosphohydrolase, ß-glucosidase, D-glucose (fermentation), D-mannose, potassium gluconate, ß-galactosidase and evenness indices of fungi were also correlated with mature stigmas. Samples of immature stigmas were influenced by cystine arylamidase, phosphatase alkaline and α-glucosidase with diversity and evenness indices of bacteria (Fig. 5b).

The bacterial and fungal profile of *P. arenaria* was different in immature and mature stigmas. The indicators used (the Simpson's dominance, Shannon diversity) presented a greater diversity of bacteria in the case of immature stigmas as compared to mature stigmas, although the values were similar (λ = 0.26 vs. 0.39 and H ‘ = 2.24 vs. 1.57). Additionally, the Pielou's evenness index for bacteria was also slightly higher (J ‘ = 0.60 vs. 0.43) in the case of immature stigmas, which proved a more even proportion of these microorganisms. Determining only two OTUs of fungal microorganisms in immature stigmas undoubtedly influenced the values of the indicators. In relation to mature stigmas, the value of the domination index was respectively higher (λ = 0.78 vs. 0.23) and the value of the diversity index was lower (H ‘ = 0.38 vs. 2.23). Interestingly, a similar value of the evenness index (J ‘ = 0.54 vs. 0.51) was obtained between immature and mature stigmas (Table 1).

**Fig. 6:**
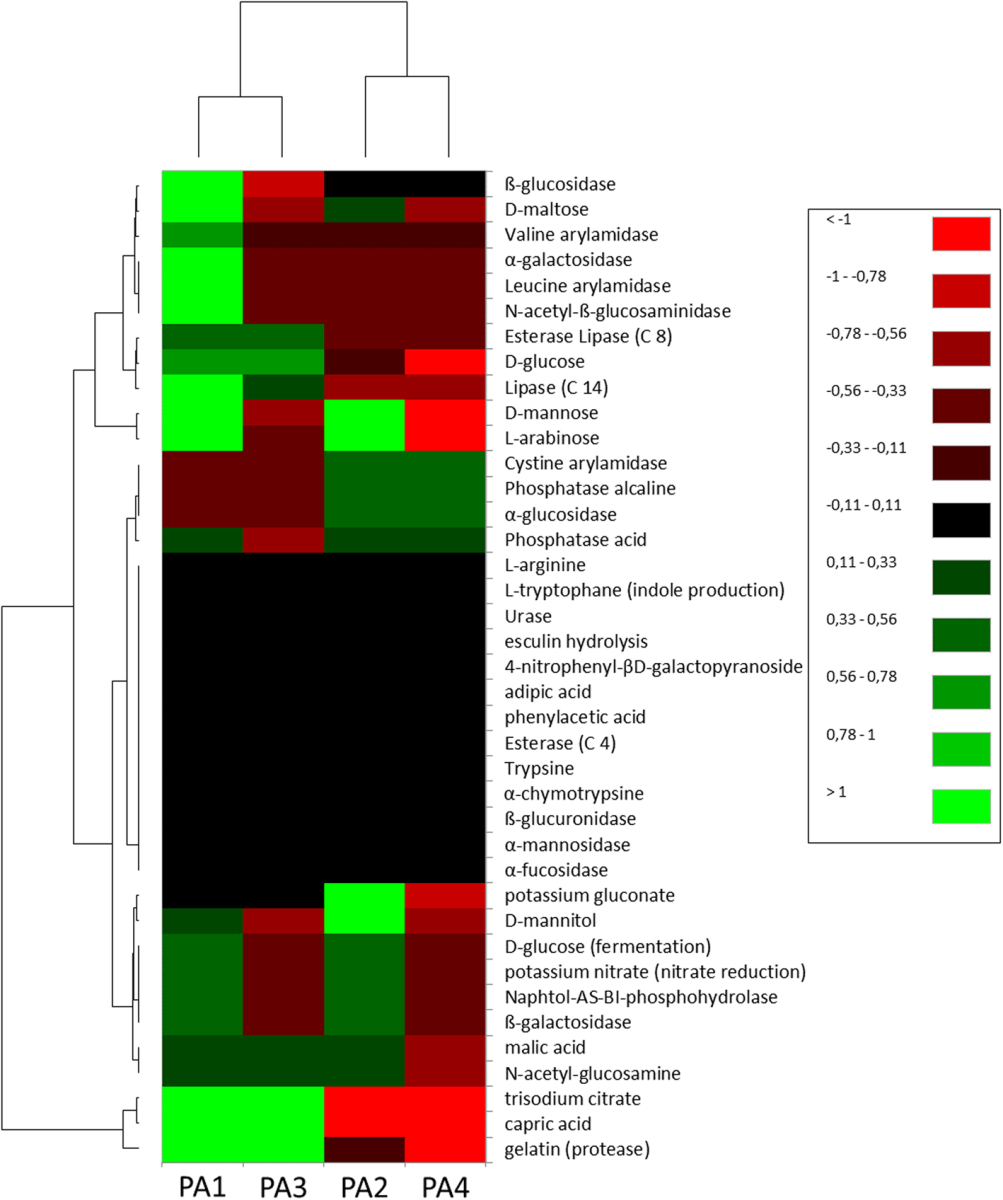
Heatmap Cluster Analysis of the normalized data: potential biochemical activity expressed by microbial communities between immature and mature stigmas samples of *Phelipanche arenaria.* The increase in the relative content of the potential biochemical activity is represented by a transition from green to black to red, as shown in the legend

Heat-map and the left hierarchical dendrogram analysis were used to compare the enzymatic activity of the bacterial communities between immature and mature stigmas samples (Fig. 6). The heat-map analysis showed differences between the samples and allowed us to separate significantly different groups. The enzymatic activity of microorganisms in mature stigmas of *P. arenaria* was different and showed more activity to that observed in immature stigmas. The conducted analysis proved that it was similar to one of mature stigmas samples (PA3), but it differed significantly in relation to the other two samples of immature stigmas (PA2 and PA4).

## Discussion

### Identification and phylogenetic characterization of isolated cultures of microorganisms

We used the traditional method, i.e., the cultivation method (isolated from plants, bacteria and fungi grown on microbiological media) and the direct method (culture independent). Both procedures generated consistent results for the most abundant taxa (in case of mature stigmas) or were partially compatible (in case of immature stigmas). Sequences classified into four genera, *Pantoea*, *Pseudomonas* and *Stenotrophomonas*, dominated the total read number (in the analysed microbial community from mature stigmas). Additionally, on culture media, these groups of bacteria grew most prolifically (Fig. 3b). However, the limitation of the culture study was significant. Only eight bacterial strains and three strains of fungi were detected on microbiological media (mature stigmas). Direct analysis of the plant material in terms of microorganism presence revealed another numerous and diverse groups of bacteria and fungi (observed in both, mature and immature). The reproducibility of the bacterial taxa detected was limited for immature stigmas tested by culture and nonculture methods. Only *Pantoea agglomerans* and *Rhodococcus corynebacterioides* were detected in culture and through next generation sequencing. No fungi were grown in laboratory culture from immature stigmas, and only two taxa were determined by direct nonculture analysis (and only in one of three samples). The culture method was insufficient to assess the microbial profile of the environmental matrix, as most bacteria and fungi did not grow on media in the laboratory [45]. It might be that the strains that grew on media in the laboratory did not constitute the dominant group realistically inhabiting the habitat under study. The most abundant strains present in the habitat likely could not grow on the media. However, our studies for mature stigmas indicate the same dominant bacterial species or genera (Table 3, Supplementary file 1). In the case of immature stigmas, there was only partial agreement. The advantage of the culture method, however, is the assessment of the viability of the isolated strains and the possibility of isolating strains for subsequent analyses or applications (e.g., *Beauveria bassiana* or *Pseudomonas* bacteria as a component of biopesticides) [46, 47].

Stigmas of *Phelipanche arenaria* were colonized by pathogenic and nonpathogenic, also potentially beneficial microorganisms. *Pseudomonas lurida* is a fluorescent species which on the one hand causes bacterial leaf spot [48] and on the other hand positively influences the growth and nutrient uptake parameters of seedlings [49]. Similar, *P. cedrina* strains has antiproliferative activity against human tumor cell lines [50] but also is recognised as bacterium-induced oxidative foliar photo-necrosis [51]. Additionally, *P. gessardii* was recently reported to improve plants' response to Pb-toxicity soils [52]. Moreover, *R. victoriana* was recognised as plant pathogen associated with acute oak decline symptoms (also *R. variigena*) [53], causing bacterial canker of *Eucalyptus* [54] but recent studies confirmed variety of beneficial features, including IAA production, phosphate solubilization, nitrogen fixation [55]. *Pantoea agglomerans* (this bacteria have been detected in immature and mature stigmas, by both methods) was described as plant pathogens and also occurs in plants as an epi- or endophytic symbiont [56]. *Bacillus megaterium* (detected only in immature stigmas) is soil bacterium of agricultural and biotechnological interest that can contribute to increasing the rate of solubilization of mineral forms of phosphorus, and induced systemic resistance and plant growth promotion [57], as well as strain A12 stimulated the growth of plants under salinity stress [58]. *Stenotrophomonas maltophilia* (detected only in mature stigmas) and *Rhodococcus corynebacterioides* (detected only in immature stigmas) as endophytic bacteria which may represent a biocontrol agent for management of bacterial wilt in plants [59, 60]. *Microbacterium testaceum* (detected only in immature stigmas) is an endophytic bacterium which helps in improving the plant growth by suppressing the growth of plant pathogens [61], and known as potassium solubilizing microorganisms [62]. *Sphingomonas aquatilis* in the rhizosphere is potentially PGPR, but it does not affect the defense against pathogens and is not a pathogen either [63].

The mature stigmas of *P. arenaria* colonize an extremely interesting fungus – *Beauveria bassiana* – that can be isolated from almost all environmental ecosystems. This microbe is an insect pathogen with a broad host range, but the pathogenicity level varies from species to species. There are numerous reports about his physiological aspects and biological control potential in insects, especially agricultural pests [46, 47, 64]. Interestingly, recent studies have shown that the activity of *B. bassiana* can promote the growth of some plants [65, 66]. Insecticidal activity is also shown by *Fusarium avenaceum*, which is recorded frequently from wheat and indicates reduced crop yield [67]. *Cladosporium subuliforme* is a representative of *C. cladosporioides* complex with *C. cladosporioides* and *C. pseudocladosporioides* which causing leaf spot of many plants [68].

### Potential functions of pathogenic, nonpathogenic and beneficial microorganisms

In this study, NGS-based ITS and 16S rRNA gene sequencing with molecular identification of cultured bacteria and fungi were combined to characterize the composition of immature and mature stigma-associated microbiota of holoparasitic plants. It should be emphasized that the analysis of the microbial communities and the structure of the stigma may be useful in research on ecological and coevolutionary adaptations between parasites and their pollinators. Anatomical, morphological and physiological differences of flowers and their parts offer a distinct habitat for colonization [6]. Connection between parasitic plants and their host allows potentially the exchange of microorganisms. It has been shown that the microbial communities of parasitic plants are derived but distinct from the host plant microbiota. Recent studies on the seed microbiome of parasitic plants have shown that some of the bacteria (*Pantoea* spp. and *Stenotrophomonas* spp.) may improve the tolerance to parasitic plants against adverse environmental conditions. These bacteria were also noted in stigmas, which may be recognised as plant growth-promoting bacteria species [42]. The presence of *Bacillus* spp. (recorded also in immature stigmas), detected in both *Cistanche armena* and *Phelipanche ramosa* seeds, was confirmed, where it was shown to be highly resistant to abiotic stress [40, 42]. Interestingly, in mature stigmas and in the floral nectar, were colonized by Enterobacteriaceae and *Pseudomonas* spp. [15] which may be important due to their structure and the presence of nutritional ingredients. These microorganisms can be endophytic or epiphytic and can be transferred by wind, rain, seeds, and pollinators [69]. The representatives of anthospheres were characterized by high diversity, but some species of *Pseudomonas* and *Acinetobacter* (Proteobacteria), *Metschnikowia* (Ascomycota), and *Cryptococcus* (Basidiomycota) were constantly recorded [11, 70], which was also confirmed by the results obtained in this study. The abundance of fungi (especially diverse population of yeast) surpassing that of bacteria in the stigma may be linked to a fertile environment with ample nutrients and optimal moisture levels conducive to fungal growth. The plant stigma exudate is an extracellular aqueous solution which is very rich in nutrients: sugars, lipids and proteins, supplemented with phenols, amino acids, reactive oxygen/nitrogen species and calcium ions. However, the sugars dominated, especially including simple sugars [71, 72]. The proteomics profiling showed even about 50 of proteins in this secreted fluid of stigma in *Lilium longiflorum* Thunb. and *Olea europaea* L. [8]. A similar content of chemical structure probably exists in plants from the Orobanchaceae family, however, more detailed research should be performed in the future. Moreover, some communities can be transmitted first and use favourable conditions, and then the composition of the microbiota will change. This situation was noted in floral nectar with yeast [14]. Additionally, the presence of secondary metabolites secreted by glandular trichomes in flowers of *Orobanche* species can also be a source of nutrients, e.g., polyphenols, lipids, polysaccharides and alkaloids for microorganisms [73, 74]. Thus, the stigma as a nutrient-rich microhabitat may create an opportunity for the development of various taxonomic and ecological groups of microorganisms.

Many microorganisms have adapted to take advantage of the stigma microhabitat of *P. arenaria*, among which one of the groups is a putative phytopathogens. It is worth emphasizing the fact that in order to accurately determine the ecological role of these microorganisms as pathogenic or beneficial for plants, it is necessary to perform detailed analyzes. The locality of fields and wastelands of *P. arenaria* involves the occurrence of different and interesting fungi associated with crops that cause many diseases, e.g., *Alternaria* spp. (black leaf spot disease), *Neoascochyta* spp. (leaf scorch on wheat), *Phoma* sp. (from leaf blight to root rot), *Puccinia graminis* (stem, black, and cereal rusts), and *Ustilago hordei* (covered smut of barley and oats). The immature stigmas were colonized only by typical plant pathogens *Alternaria* sp. and *Blumeria* sp.. Some of these pathogens are especially dangerous because attack plants during the pollination process, like *Metschnikowia cibodasensis* and *Microbotryum saponariae* which can replacing pollen by fungal spores leading to sterilize their host plants [75]. *Gibberella tricincta* infects plants beginning at anthesis, and this results in dried prematurely of plants or the production of damaged seeds [76]. The stigmas were also colonised by the rarer pathogens, *A. eichhorniae* which the bioherbicidal properties are used against *Eichhornia crassipes*, one of the world's worst aquatic weeds [77]. Additionally, the presence of typical plant pathogens may cause the plant to be attacked more frequently by other microorganisms such as *Cladosporium* spp., especially *Mycosphaerella tassiana* (anomorph: *Cladosporium herbarum*). However, these microorganisms also are known antagonists to other pathogens [78]. *Acremonium implicatum* and *Sarocladium strictum* known as plant pathogens, but very often neutrally inhabiting plants or being endosymbionts with insecticidal properties – widely studied as natural biopreparations for protection against plant pests. Some of them also have the potential to protect the host against pathogenic fungi [79, 80].

Apart from phytopathogens in the stigma of *Phelipanche arenaria*, there are also microorganisms belonging to the plant growth-promoting group. *Serratia marcescens* can influence seedling growth in wheat and some medical plants. It is also described as a rice endophyte [81–83]. Additionally, numerous plant growth-promoting bacteria, which are also rhizosphere competent, e.g.: *Acinetobacter rhizosphaerae*, *Chryseobacterium* sp., and *Luteibacter* sp. were recognized. In addition to increasing plant growth, these bacteria improve the nutrient status of soil and the availability of nutrients to plants [84]. The presence of *A. rhizosphaerae* was detected in the cold deserts of the Himalayas [85]. The beneficial bacterium from the rhizosphere is *Stenotrophomonas rhizophila*, which indicates plant growth when soil salinity is high [86]. Furthermore, there are also rare species known mainly from rhizospheric soil of grasses from land in northern Spain, such as the phosphate-solubilizing bacterium *Pseudomonas rhizosphaerae.* [87]. The community of rhizosphere-related microorganisms is extremely diverse, including representatives of *Pseudarthrobacter* sp. which extracts showed the antibacterial activity against plant pathogens, and antioxidant activity (high phenol and flavonoid content in the extracts of plants treated with this strain) [88]. Some bacteria have potential to grow in nutrient-poor and extreme environments, i.e., *Methylobacterium* species utilize reduced carbon compounds, such as methanol and methane [89]. *Massilia* species have plant growth-promoting traits and biocontrol activity, as well as have ability to degrade organic compounds [90]. It should also be noted that besides rhizosphere microbes, there are also some of the microorganisms which improving seed germination and seedling growth. *Pseudomonas* spp., *Bacillus* spp., *Enterobacter* spp., *Pantoea* spp. and *Stenotrophomonas* spp. exhibit these properties in seed treatment [42, 91]. Several types of yeasts and some of these are also endophytes were isolated, e.g.: *Aureobasidium pullulans*, *Bullera* spp., *Candida elateridarum*, *Cryptococcus uniguttulatus*, *Filobasidium* spp., *Hannaella coprosmae*. The abundance of yeast is related to the presence of nutrients in the floral nectar [14, 18]. Likewise, stigmas, which are also rich in these substances, hence their abundance may occur [71, 72]. Additionally, even using *Vishniacozyma victoriae* as yeast biocontrol contributes to significant effectiveness against the causal agents of disease of pear fruits [92] or *Aureobasidium pullulans* for control of postharvest decay of pear [93]. Moreover, *A. pullulans* with a worldwide distribution is recognized as an endosymbiont without causing any symptoms of disease. This yeast-like fungus produces a wide range of natural products that are well known and have been described as beneficial for biotechnological and environmental applications (e.g., production of different enzymes, antimicrobial compounds) [94, 95]. More research is needed to accurately determine the role of ecologically diverse microbes inhabiting the stigmas of *P. arenaria*.

### Relationships between identified microorganisms, diversity indices and their biochemical potential

The most important relationships between identified microbiota and diversity indices, as well as between the biochemical potential of identified microorganisms and diversity indices were shown in Fig. 5. The fungal profile of mature stigmas was extremely rich compared to the number of OTUs found in immature stigmas and to the number of bacterial OTUs found in both immature and mature stigmas (Table 1, Fig. 5a). Bacterial communities colonizing immature *Phelipanche arenaria* stigmas showed greater diversity and contained 21 unique OTUs that were not found in mature stigmas but we identified only two fungal genera – *Alternaria* and *Blumeria* which are typical plant pathogens (Table 2, Fig. 5a). In contrast, mature stigmas were associated with more enzymatic activity of the bacteria (Fig. 6). The stigmas of *P. arenaria* were colonized by microorganisms with a wide range of activity from microorganisms that were potentially beneficial to those that were pathogenic to plants. The mature stigmas were positively correlated with most of identified fungi, and *Stenotrophomonas* spp., *Pseudomonas* spp., *P. fragi*, *Pantoea ananatis*, *Xanthomonadaceae*, *Rhizobiaceae*, *Pedobacter* spp. *Paenibacillus* spp., as well as the OTUs and diversity indices of fungi. During the short period of development of stigmas, a number of dynamic interactions occur, especially competition between the microorganisms that inhabit them. Some pathogenic bacteria or fungi observed in stigma samples may be suppressed by microorganisms capable of biocontrol. In stigmas mature, beneficial bacteria are less important, while numerous fungi have the ability to act against plant pathogens. Immature stigmas of *P. arenaria* were colonized mainly by bacteria and the fungi had lesser importance in contrast to mature stigmas, where 80 OTUs of fungi have been identified. The rhizosphere-associated bacteria and fungi were also often found in stigmas, especially in immature stigmas, which may be due to the specific biology, including often several years of underground life. Moreover, *P. arenaria* populations occupy usually open and relatively level areas sparsely vegetated in dry, alkaline and sandy soils on gently inclined slopes which facilitates the colonization of this group of bacteria. In addition, compared to other species of Orobanchaceae, this population in the studied area reached a small height of 8 to 20 cm [96, 97].

Bacteria identified in mature stigmas showed positive or negative correlations for most biochemical enzymes. These microorganisms were able to produce many enzymes involved in the degradation of plants cells: β-galactosidase, α-glucosidase, protease in immature stigmas, and α-galactosidase, β-galactosidase, β-glucosidase, protease in mature stigmas. Moreover, identified bacteria in both immature and mature stigmas use capric, malic, phosphatase acids as a source of substances from the decomposition of organic matter in order to provide energy. The results obtained indicate the wide range of enzymatic activity of bacteria (more and stronger activity in mature stigmas) which the utilization of lipids (Esterase Lipase (C8), Lipase (C14)), saccharides (D-maltose, D-glucose, D-mannose, L-arabinose), proteins (valine arylamidase, leucine arylamidase, cystine arylamidase), and can flexibly adapt to environmental conditions. These bacteria were able to nitrate reduction, and were characterized by activity of naphthol-AS-BI-phosphohydrolase, as well as phosphatase alkaline and potassium gluconate (only immature stigmas) (Fig. 6). *Pantoea ananatis*, *Stenotrophomonas* spp., *Pseudomonas fragi*, *Pedobacter* spp., Xanthomonadaceae, Rhizobiaceae, *Paenibacillus* spp., clear correlated with mature stigmas may able to decomposing glycosyl hydrolases (α-galactosidase) and amino acids (valine arylamidase, leucine arylamidase) (Figs. 5 and 6). Interestingly, the activity of N-acetyl-glucosamine only identified in bacteria of mature stigmas will be responsible for the degradation of chitin which can lead to the destruction of fungi or insect pathogens [98]. Moreover, N-acetyl-ß-glucosaminidase in immature and mature stigmas was associated with the use of chitin by the microbial communities. The biochemical activity of immature stigmas (PA3) was similar to mature stigmas samples (PA1), due to the presence of Enterobacteriaceae and *Stenotrophomonas* spp. (PA3), which were not found in other immature stigmas samples (PA2 and PA4). In addition, this stigma sample (PA3) had higher frequency of *Rhodococcus corynebacterioides* than other immature stigma samples (PA2 and PA4), also we observed higher abundance of *Serratia* spp., which was not found in the other samples tested. The immature stigmas (PA2) were clear correlated positively with several bacterial genera and only one fungal genus – *Blumeria*, as well as diversity and evenness indices of bacteria. *Sphingomonas* spp., *Methylobacterium-Methylorubrum* sp., *Xylophilus* sp., *Chryseobacterium* spp., *Massilia* sp., *Pseudarthrobacter* sp., *Luteibacter* spp., *Variovorax* sp., *Aureimonas* spp. may have a biochemical potential with activity of α-glucosidase and cystine arylamidase, and phosphatase alkaline (Figs. 5 and 6).

## Conclusions

Our analysis is the first microbiota study of both the stigma-associated bacteria and fungi of *Phelipanche arenaria* using culture-dependent methods with gene-targeted next-generation sequencing. These results showed unexpected diversity in the stigma of the *P. arenaria* microbiota, including 39 to over 51 OTUs per sample for bacterial OTUs, as well as 80 OTUs in fungal communities.

The microbial profile of the studied stigma of parasitic plant is formed by putative beneficial, nonpathogenic and pathogenic bacteria, and fungi may potentially play a key role in promoting or suppressing many processes associated with the growth, reproduction and health of plants. Interestingly, in the bacterial profile, we identified many bacteria associated with the rhizosphere. However, yeasts were commonly noted among fungi. Enzymatic activity of microorganisms in mature stigmas of *P. arenaria* was more strongly observed than that in immature stigmas. These stigma interactions, which are determined by bacterial and fungal communities, can be one the most important ecological microbial communities of this small but diverse microhabitat. A more detailed examination about microbiota of parasitic plants can explain the structure and diversity of microorganisms as dominant samples or as different points in a temporal dynamic. However, a better understanding is needed on how the evolution of the microbial communities of the flower stigma develops and what role these microorganisms can play in the biology of such rare and endangered parasitic plants.

## Methods

### Plant material and sample collection

*Phelipanche arenaria* and other broomrape species become visible above the surface of the soil only at the time of flowering. The distribution of *P. arenaria* has been reported in northwest Africa and western, central, southern and eastern Europe to the Caucasus, Asia Minor and Central Asia [37]. *P. arenaria* is a critically endangered species in Germany, Poland, as well as endangered in Czech Republic and Slovakia [99]. In Poland, populations consist of a small number of individuals, and the species occurs only in a few localities in Lower Silesia, Middle Vistula Gap, Częstochowa Upland and the Małopolska Upland [99, 100]. Polish localities constitute the northern limit of its range [97]. The species parasitizes the roots of *Artemisia* sp., mainly *A. campestris* L. (Asteraceae) [101]. This parasitic plant is relatively small, usually 20 cm tall, and forms short and scaly simple stems that end with violet or bluish flowers. Inflorescence takes up three-quarters of emerged stems. The flowers are bisexual, zygomorphic, and insect pollinated. The flowers remain constantly open and the length of flowering lasts from a few to several days. The stigma in *P. arenaria* flowers comprises two lobes that are whitish with a contrasting colouration and shine to the corolla, constituting specific adaptations of flowers for insect pollination [37, 96, 102].

**Fig. 7:**
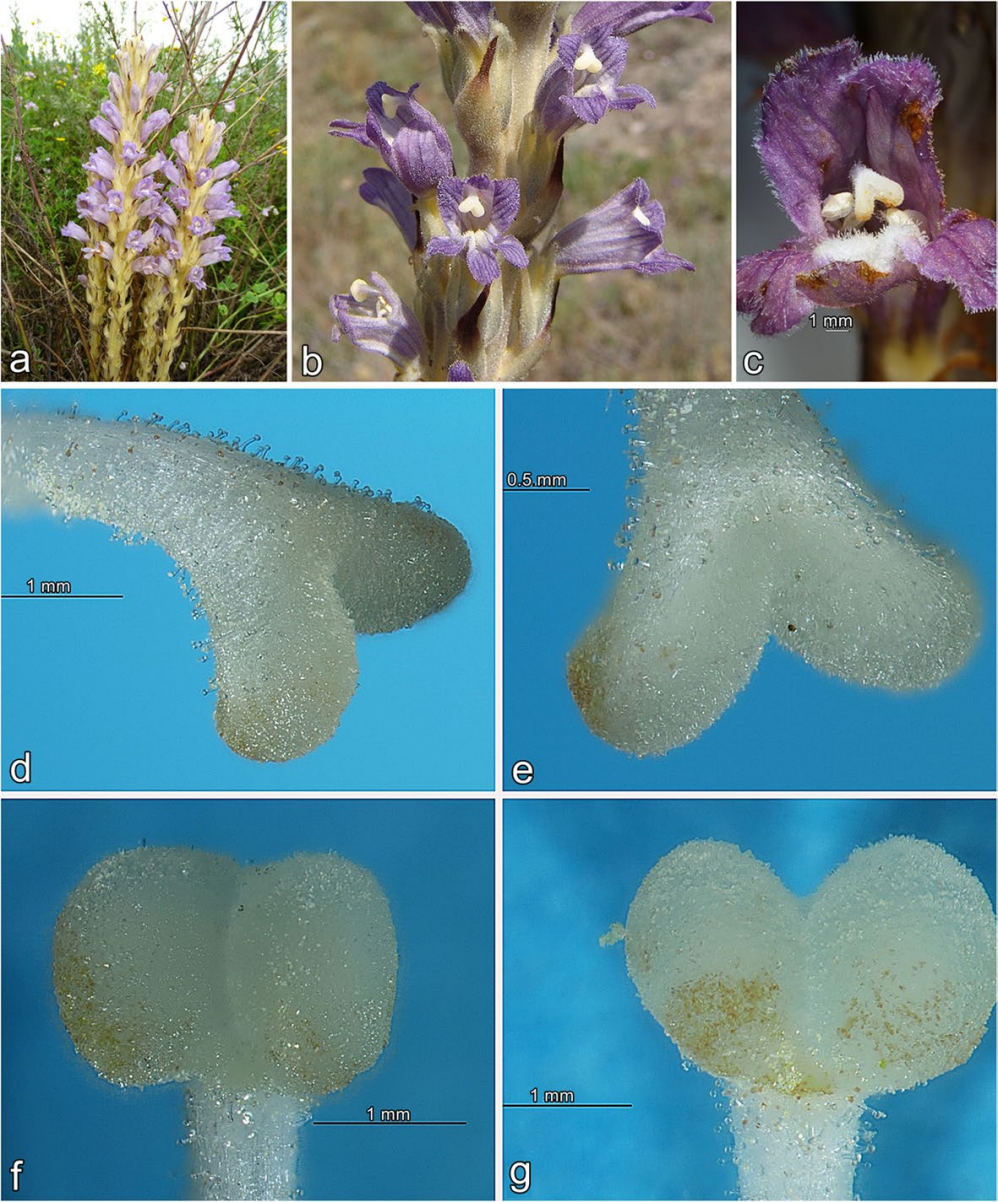
Studied holoparasitic *Phelipanche arenaria*. **a** general habit, (**b**, **c**) opened flower with whitish stigma, phot. R. Piwowarczyk; (**d-g** ZOOM micrographs of two-lobed stigma of *P. arenaria* with numerous papillae covered with a viscous secretion and above the pistil with glandular trichomes, phot. K. Zubek. Scale bars: **c**, **d**, **f**, **g** 1 mm; **e** 0.5 mm

We collected stigma samples of *Phelipanche arenaria* from Zwierzyniec near Szaniec in the Małopolska Upland in Poland in June 2021. The parasite grows on sandy fallows, in the vicinity of cultivated fields, excavation of sand and pine forests, sparsely overgrown by *Pinus sylvestris* L. with admixture species from *Festuco-Brometea*, *Molinio-Arrhenatheretea* and *Stellarietea mediae* classes, and parasitized *A. campestris* (Fig. 7a). A phytosociological relevance of all accompanying species is available in Piwowarczyk and Przemyski [97]. *P. arenaria* is a critically endangered species, as well as partially protected in Poland, and only a limited number of individuals (10) have been authorized to conduct research in this project. The plant materials were identified by Renata Piwowarczyk. Experimental research and field studies on plants, including the collection of plant material was complied with relevant institutional, national, and international guidelines and legislation, and necessary permits were obtained. Material sampling was done with permission no. WPN.I.6400.3.1.2021.AD from the Regional Directors for Environmental Protection in Poland. For our research we chose a population from Zwierzyniec which is one of the most numerous in Poland. Samples of *P*. *arenaria* with closed petal flowers (immature stigmas) and fully opened (mature stigmas) were selected. The number of stigmas that was collected on each individual varied. It can range from 5 to over 25 stigmas. About 300 immature and mature stigmas (150 stigmas per sample) were immediately dissected sterile from the flowers into DNA-free sterile plastic tubes stored at 4 ± 0.5 °C until analysis, which was carried out within 48 h. For culture-dependent and culture-independent methods, 1 g each of stigmas samples were analysed. For these samples, the pistil was cut as close as possible to the base of the stigma (Fig. 7b–g). The voucher specimen was stored in the Herbarium of Jan Kochanowski University in Kielce (KTC), Poland [103]. The collection is not numbered, but the herbarium sheets are stored in a separate section under the name “Parasitic plants”, and the species are sorted alphabetically, in boxes labeled with the species name. The plant names were updated based on the World Flora Online (WFO) [104].

### Isolation and identification of microorganism inhabits plant tissue

#### Isolation of microorganisms from plant tissue cultures

**Fig. 8:**
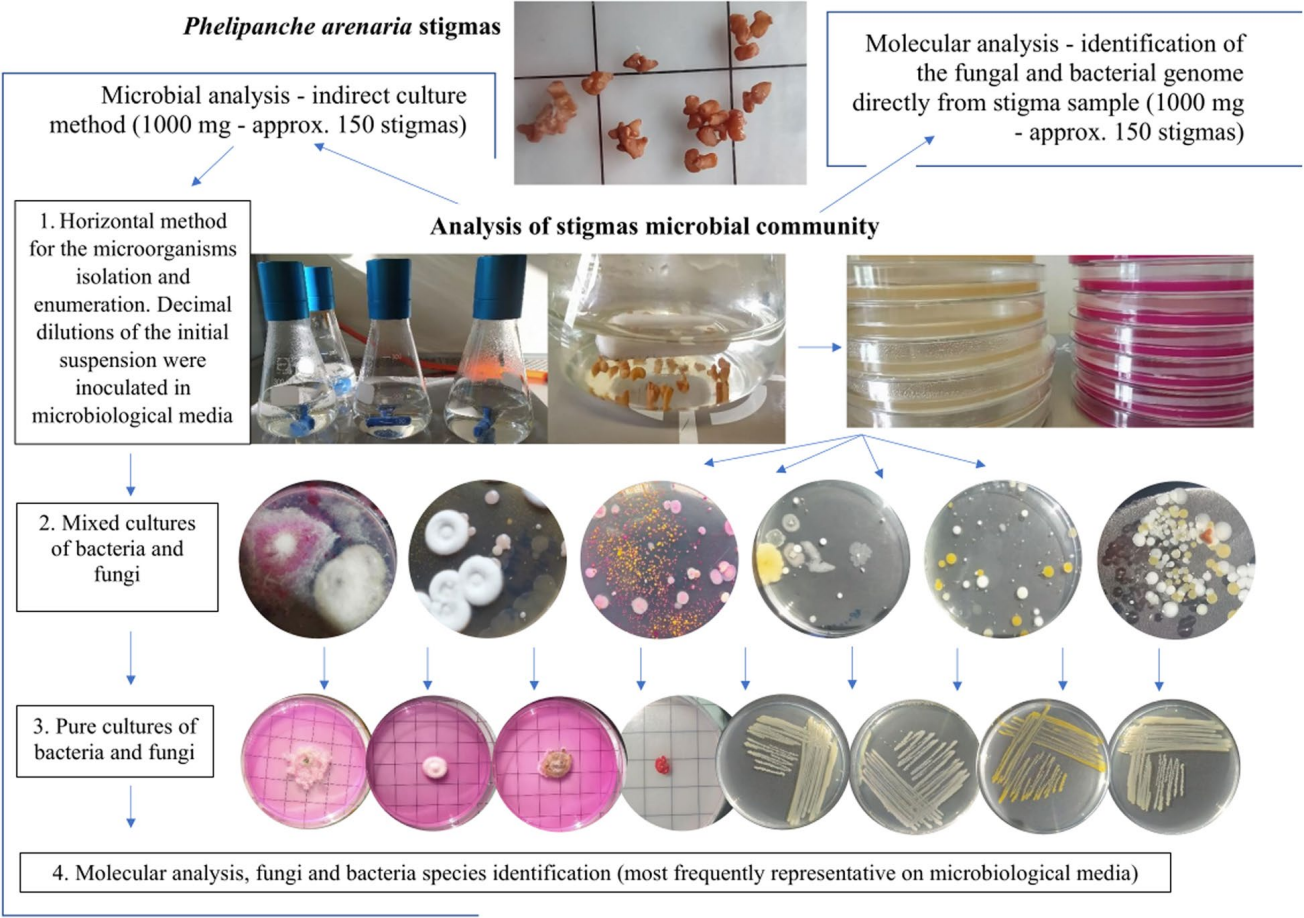
Experiment scheme of analysis microbial communities of stigmas in *Phelipanche arenaria*

The immature and mature stigmas microbiota was studied by the culturing method using microbiological media and by the non-culturing method extracting bacterial and fungal DNA directly from plant samples (Fig. 8). Horizontal method for the isolation and enumeration of yeasts, moulds, bacteria was used. The microbial analysis was based on the European Standard ISO (ISO 4833–1) [105]. One sample of mature stigmas and 3 samples of immature stigmas were used for the study. A sample (1 g, approx. 150 stigmas) was divided into 3 equal parts. 10 stigmas were taken from each part and placed directly in the warm sterile medium. Remaining stigmas were placed in saline (0.85% NaCl aqueous solution) and suspension dilutions were made. Dilutions of the stigmas containing suspension to 10^–10^ were made. Dilutions were made for each of the 3 parts separately. Stigmas were inserted into flasks with sterile physiological fluid and mixed to precipitate the microorganisms from the stigmas into the liquid phase of the solution (shaken for 30 min at 200 rpm). A series of dilutions of the stock solution was made. One milliliter of each dilution was withdrawn and placed into sterile Petri dishes (in triplicate for each dilution and group of microorganisms). Then, the content of the plates was covered with microbiological media. Cultures were incubated for 3–5 (bacteria) and 7 (fungi) days at 30 °C. Pure cultures were obtained from a mixed culture. For molecular analysis, morphologically different isolates grown on media were selected (microscopic observation of colonies and cells—microscopic preparations). The obtained inoculates were subjected to molecular analysis to identify the species (further description – molecular analysis). Additionally, the number of bacteria and fungi inhabiting the stigmas was determined. For this purpose, the colonies of bacteria and mould were counted according to the formula: average number of colonies/dilution used (log cfu ·g^−1^ ± SD stigmas). Colonies were counted using an automatic colony counter (Alchem PCC04). Three microbiological media were used: for bacteria – Trypticase Soy Agar (BioMaxima Poland), for yeast – Yeast Extract-Peptone-Glycerol Medium (BTL Poland), and mould fungi – Rose Bengal Agar (BTL Poland [105–108].

#### Identification and phylogenetic analysis of 16S rRNA and ITS from pure cultures of bacteria and fungi

Genomic DNA was extracted using a commercial Genomic Mini AX Bacteria + kit (A&A Biotechnology, Poland) with an additional step of mechanical lysis in a FastPrep-24 (MP Biomedicals, United States) with zirconia beads. Amplification of the 16S rRNA fragment was performed using a forward 27F primer: 5’GAG TTT GAT CCT GGC TCA G 3’ and reverse 1492R primer: 5’ACG GCT ACC TTA CGA CTT 3’. Amplification of the internal transcribed spacer (ITS) fragment was carried out using ITS1 primer: 5’TTC GTA GGT GAA CCT GCG G 3’ and ITS4 primer: 5’TCC TCC GCT TAT TGA TAT GC 3’. Amplification was carried out in a 50 μl reaction volume containing 25 μl of PCR Mix Plus HGC (A&A Biotechnology, Poland), 0.2 µl of 100 µM forward and reverse primers, and 5 μl of DNA extract. The PCR conditions were as follows: 120 s at 94 °C of initial denaturation, 30 cycles of denaturing at 94 °C for 30 s, primer annealing for 45 s at 58 °C (for 16S rRNA) and 52 °C (for ITS), primer extension at 72 °C for 90 s, and a final extension step for 300 s at 72 °C. PCR products were purified using Clean Up (A&A Biotechnology, Poland), suspended in 10 mM Tris–HCl pH 8.0 buffer and diluted to 50 ng/µl. The sequencing was conducted by Macrogen (The Netherlands). The associated sequences are available from the GenBank database at accession numbers OQ132617-OQ132625, MW731567-MW731574 and MW849231-MW849233.

### Identification and properties of microbiota inhabitant plant tissue

#### Illumina library preparation and sequencing

Genomic DNA was extracted from pooled stigma tissue samples (1000 mg) using a commercial Genomic Mini AX Bacteria + kit (A&A Biotechnology, Poland) with an additional step of mechanical lysis in a FastPrep-24 (MP Biomedicals, United States) with zirconia beads. DNA was purified using an Anty-Inhibitor Kit (A&A Biotechnology, Poland), and DNA quantity was measured using a Qubit 4 Fluorometer. The presence of bacterial DNA in the tested sample was confirmed with real-time PCR carried out in an Mx3000P thermocycler (Stratagene, United States) using SYBR Green dye as a fluorochrome and universal starters [109].

The V3-V4 16S rRNA and ITS amplicon libraries were prepared for the sample in accordance with 16S Metagenomic Sequencing Library Guidelines Preparation Part # 15,044,223 Rev. B, Two-step PCR with a Herculase II Fusion DNA Plymerase Nextera XT Index Kit V2 kit. The gene fragments were amplified with the PCR primers recommended for the Illumina technique. Primers ITS3F (GCATCGATGAAGAACGCAGC) and ITS4R (TCCTCCGCTTATTGATATGC) for fungal ITS library, 341F (CCTACGGGNGGCWGCAG) and 805R (GACTACHVGGGTATCTAATCC) for bacterial 16S rRNA libraries were employed. The quality of the library was checked according to the Illumina qPCR Quantification Protocol Guide using the 2200 TapeStation System (Agilent, United States). Finally, sequencing was performed on an Illumina MiSeq PE300 platform. Each file underwent quality control (QC), which included quality filtering (removing sequences with ≥ 5 ambiguous base pairs) and length filtering (removing sequences with a length ≥ 2 standard deviations from the mean). Sequences below three reads (singletons) or with abundance less than 0.0005% were removed after generating the ASV table. The bacterial and fungal NGS sequences of stigmas were submitted to the NCBI Short Reads Archive (SRA) under the project number PRJNA714256 and MG-RAST under project number mgp105405. The results were obtained using QIIME 2 [110] and Pipeline based on the SILVA [111] or UNITE [112] databases. NGS library preparation and amplicons sequencing was conducted by Macrogen (The Netherlands).

#### Biochemical potential of microbial communities inhabiting stigmas

The plant homogenizate was diluted in 1 mL of sterile physiological saline (NaCl 0.85%). Before the biochemical tests, prepared suspension was transferred to peptone water mixed with 1% TSB (Tryptic soy broth, Merck, Germany) and incubated overnight at 28 °C. After overnight incubation, the pre-cultures were diluted to the appropriate optical density (A = 0.1 or 0.8), depending on the test, and applied to the API NE and API ZYM test kits according to the instructions provided by the producer (Biomerieux, France). Assay kits were incubated at 28 °C for 24 h in the case of API 20 NE and 4 h in the case of API ZYM [113].

### Phylogenetics and calculations

#### Phylogenetic analysis

All obtained sequences 16S rRNA and ITS of bacteria and fungi from culture method were verified against the NCBI-GenBank database (National Center of Biotechnology Information, USA) using the BLAST algorithm [114]. The sequence similarity to the most probable species is shown in the Supplementary file 1. In order to visualize phylogenetic relationships, three each of the most similar sequences to the obtained amplicons and the sequences with a lower degree of relatedness were collected, and the MEGA X [115] was used for these analyses. For alignment and taxonomic allocation of all 16S rRNA and ITS sequences following parameters were used: ClustalW algorithm with gap opening penalty 15 and gap extension penalty seven for pairwise alignment. Thus, an alignment matrix of 1332 nucleotides with gaps for bacteria and 533 nucleotides with gaps for fungi was obtained. In order to select a method for the reconstruction of phylogenetic relationships between strains used to construct a Maximum-Likelihood tree as determined using the “Find best DNA/Proteins model (ML)” tool in the software. Bootstrap values were inferred from 1,000 replicates and percentages displayed next to the relevant branch. For the 16S rRNA, the most optimal BIC (Bayesian Information Criterion) was selected for the Tamura 3-parameter model, taking into account the discrete gamma distribution (+ G) for non-uniform evolution rate and evolutionary invariant sites (+ I). However, for the ITS sequence, Kimura 2-parameter was chosen taking into account the discrete gamma distribution (+ G) for non-uniform evolution rates [115, 116] (Figs. 1 and 2).

#### Statistical calculations

The Simpson dominance (λ), Shannon diversity index (H’), and Pielou’s evenness index (J’) [117–119] determined the OTU compositions of bacteria and fungi of each sample. Moreover, according to the paper of Przemieniecki et al., [120] for bacteria and Kurowski et al., [121] for fungi, the domination classes of OTUs were analysed. Principal component analysis (PCA) and agglomerative hierarchical clustering (AHC) used to calculate general relationships in the XLSTAT program. Correlation matrices for principal component analysis were estimated using Pearson's correlation coefficient. For AHC, the Bray–Curtis test was used with Ward’s agglomerative method. Two sided Fisher’s exact test, with the Newcombe-Wilson confidence interval method was used to compare the significance of the relative proportion difference in taxonomic distribution of samples. Results with *q* < 0.05 were considered significant and the unclassified reads were removed from analyses. The Heatmap matrix for the biochemical potential of microbial communities was made using the formula within the data for the tested biochemical feature (and not within the features of the tested object) according to the formula—(x-mean)/ standard deviation.

### Supplementary Information

**Additional file 1: Supplemental Table 1.** The sequence similarity to the most probable species of bacteria and fungi identified in culture method colonising stigmas of *Phelipanche arenaria*.

## Data Availability

The bacterial and fungal NGS sequences of mature stigmas were submitted to the NCBI Short Reads Archive (SRA) under the project number PRJNA714256 (https://www.ncbi.nlm.nih.gov/bioproject/PRJNA714256) and MG-RAST under project number mgp105405 (https://www.mg-rast.org/mgmain.html?mgpage=search&search=mgp105405), and all the associated sequences are available from the GenBank database at accession numbers OQ132617-OQ132625, MW731567-MW731574 and MW849231-MW849233.
