# Supplementary material for: Stigmas of holoparasitic Phelipanche arenaria (Orobanchaceae) – a suitable ephemeric flower habitat for development unique microbiome
Source: BMC Plant Biol. 2023 Oct 11;23:486. doi: 10.1186/s12870-023-04488-1 (PMC10566107; doi:10.1186/s12870-023-04488-1)
Supplement: Supplementary file 1 — Additional file 1: Supplemental Table 1. The sequence similarity to the most probable species of bacteria and fungi identified in culture method colonising stigmas of Phelipanche arenaria. [file 12870_2023_4488_MOESM1_ESM.docx]

**Stigmas of holoparasitic *Phelipanche arenaria* (Orobanchaceae) – a suitable ephemeric flower habitat for development unique microbiome**

Karolina Ruraż^a,*^, Sebastian Wojciech Przemieniecki^b^, Magdalena Błaszak^c^, Sylwia Dagmara Czarnomska^d^, Ireneusz Ochmian^e^, Renata Piwowarczyk^a^

^a^Center for Research and Conservation of Biodiversity, Department of Environmental Biology, Institute of Biology, Jan Kochanowski University, Uniwersytecka 7, PL-25-406, Kielce, Poland; karolina.ruraz@ujk.edu.pl, ORCID: 0000-0003-4831-7712; piwowarczyk@ujk.edu.pl, ORCID: 0000-0003-0507-7835

^b^Department of Entomology, Phytopathology and Molecular Diagnostics, University of Warmia and Mazury in Olsztyn, Prawocheńskiego 17, PL-10-720 Olsztyn, Poland; sebastian.przemieniecki@uwm.edu.pl, ORCID: 0000-0003-1222-0310

^c^Department of Bioengineering, West Pomeranian University of Technology Szczecin, Słowackiego 17, PL-71-434 Szczecin, Poland; magdalena.blaszak@zut.edu.pl, ORCID: 0000-0001-6798-4639

^d^Museum and Institute of Zoology, Polish Academy of Sciences, Nadwiślańska 108, PL-80-680 Gdańsk, Poland; s.czarnomska@gmail.com, ORCID: 0000-0002-8081-0956

^e^Department of Horticulture, West Pomeranian University of Technology Szczecin, Słowackiego 17 Street, PL-71-434 Szczecin, Poland; iochmian@zut.edu.pl, ORCID: 0000-0002-3606-1927

*Corresponding author

Center for Research and Conservation of Biodiversity, Department of Environmental Biology, Institute of Biology, Jan Kochanowski University, Uniwersytecka 7, PL-25-406 Kielce, Poland; e-mail address: karolina.ruraz@ujk.edu.pl

Supplementary file 1

Supplemental Table 1 The sequence similarity to the most probable species of bacteria and fungi identified in culture method colonising stigmas of *Phelipanche arenaria*

| Accession |  | | Query Cover (%) | Per. Ident (%) |
| --- | --- | --- | --- | --- |
| Immature stigmas | | | | |
| OQ132617 | PAB9 | *Bacillus megaterium* | 100 | 99.51 |
| OQ132618 | PAB10 | *Microbacterium testaceum* | 100 | 100 |
| OQ132619 | PAB11 | *Pantoea agglomerans* | 100 | 99.86 |
| OQ132620 | PAB12 | *P. agglomerans* | 100 | 100 |
| OQ132621 | PAB13 | *Rahnella variigena* | 100 | 100 |
| OQ132622 | PAB14 | *R. victoriana* | 99 | 99.79 |
| OQ132623 | PAB15 | *R. victoriana* | 99 | 99.79 |
| OQ132624 | PAB16 | *Rhodococcus corynebacterioides* | 99 | 99.81 |
| OQ132625 | PAB17 | *R. corynebacterioides* | 99 | 99.93 |
| Mature stigmas | | | | |
| MW731572 | PAB6 | *P. agglomerans* | 100 | 99.62 |
| MW731571 | PAB5 | *Pseudomonas cedrina* | 100 | 99.31 |
| MW731569 | PAB3 | *P. gessardii* | 100 | 99.84 |
| MW731573 | PAB7 | *P. lurida* | 100 | 99.69 |
| MW731567 | PAB1 | *Sphingomonas aquatilis* | 100 | 100 |
| MW731568 | PAB2 | *Stenotrophomonas maltophilia* | 100 | 99.69 |
| MW731570 | PAB4 | *S. maltophilia* | 100 | 98.84 |
| MW731574 | PAB8 | *S. maltophilia* | 100 | 99.55 |
| MW849231 | PAF1 | *Beauveria bassiana* | 99 | 100 |
| MW849233 | PAF4 | *Cladosporium subuliforme* | 99 | 100 |
| MW849232 | PAF2 | *Fusarium avenaceum* | 100 | 99.81 |
